# Paraneoplastic encephalomyeloradiculits with multiple autoantibodies against ITPR-1, GFAP and MOG: case report and literature review

**DOI:** 10.1186/s42466-021-00145-w

**Published:** 2021-10-11

**Authors:** Anna Cirkel, Klaus-Peter Wandinger, Claudia Ditz, Jan Leppert, Lars Hanker, Christoph Cirkel, Alexander Neumann, Jan Brocke, Romana Höftberger, Lars Komorowski, Sven Perner, Frank Leypoldt, Tobias Wagner-Altendorf, Thomas F. Münte, Georg Royl

**Affiliations:** 1 Department of Neurology, University Hospital of Schleswig-Holstein Lübeck, Lübeck, Germany; 2 Institute of Clinical Chemistry, University Hospital of Schleswig-Holstein, Kiel, Germany; 3 Department of Neurosurgery, University Hospital of Schleswig-Holstein Lübeck, Lübeck, Germany; 4 Department of Gynecology, University Hospital of Schleswig-Holstein Lübeck, Lübeck, Germany; 5 Department of Neuroradiology, University Hospital of Schleswig-Holstein Lübeck, Lübeck, Germany; 6 Neurological Rehabilitation Center, Segeberger Kliniken, Bad Segeberg, Germany; 7 Institute of Neurology, Medical University Vienna, Vienna, Austria; 8 Institute of Experimental Immunology, Euroimmun AG, Lübeck, Germany; 9 Department of Pathology, University Hospital of Schleswig-Holstein Lübeck, Lübeck, Germany; 10 Research Center Borstel, Leibniz Lung Center, 23538 Lübeck and, 23845 Borstel, Germany; 11 Department of Neurology, University Hospital of Schleswig-Holstein Kiel, Kiel, Germany; 12 Institute of Psychology II, University Hospital of Schleswig-Holstein, Lübeck, Germany

**Keywords:** Paraneoplastic, ITPR-1, GFAP, MOG, Autoantibody, Encephalitis, Encephalomyelitis, Encephalomyeloradiculits, Multiple antibodies

## Abstract

**Background:**

Recently, antibodies against the alpha isoform of the glial-fibrillary-acidic-protein (GFAPα) were identified in a small series of patients with encephalomyelitis. Coexisting autoantibodies (NMDA receptor, GAD65 antibodies) have been described in a few of these patients. We describe a patient with rapidly progressive encephalomyeloradiculitis and a combination of anti-ITPR1, anti-GFAP and anti-MOG antibodies.

**Case presentation and literature review:**

A 44-year old caucasian woman with a flu-like prodrome presented with meningism, progressive cerebellar signs and autonomic symptoms, areflexia, quadriplegia and respiratory insufficiency. MRI showed diffuse bilateral T2w-hyperintense brain lesions in the cortex, white matter, the corpus callosum as well as a longitudinal lesion of the medulla oblongata and the entire spinal cord. Anti-ITPR1, anti-GFAP and anti-MOG antibodies were detected in cerebrospinal fluid along with lymphocytic pleocytosis. Borderline tumor of the ovary was diagnosed. Thus, the disease of the patient was deemed to be paraneoplastic. The patient was treated by surgical removal of tumor, steroids, immunoglobulins, plasma exchange and rituximab. Four months after presentation, the patient was still tetraplegic, reacted with mimic expressions to pain or touch and could phonate solitary vowels. An extensive literature research was performed.

**Conclusion:**

Our case and the literature review illustrate that multiple glial and neuronal autoantibodies can co-occur, that points to a paraneoplastic etiology, above all ovarian teratoma or thymoma. Clinical manifestation can be a mixture of typically associated syndromes, e.g. ataxia associated with anti-ITPR1 antibodies, encephalomyelitis with anti-GFAPα antibodies and longitudinal extensive myelitis with anti-MOG antibodies.

**Supplementary Information:**

The online version contains supplementary material available at 10.1186/s42466-021-00145-w.

## Background

Recently, antibodies against the alpha isoform of glial fibrillary acidic protein (GFAPα) have been found in a group of patients with immunotherapy responsive relapsing autoimmune meningoencephalomyelitis [6, 7]. Myelin oligodendrocyte glycoprotein (MOG) antibodies are most often found in relapsing optic neuritis or neuromyelitis optica spectrums diseases, acute demyelinating encephalomyelitis (ADEM) and in a few cases with encephalitis, complicated by respiratory impairment [8, 9]. A few cases with autoimmune cerebellar ataxia and peripheral neuropathy targeting inositol 1,4,5-triphosphate receptor type 1 (ITPR1) have been published [3, 8, 9]. Some cases showed a systemic cancer expressing onconeural antigens leading to paraneoplastic encephalitis [8, 9]. We describe a patient with severe meningoencephalomyeloradiculitis and a combination of anti-ITPR1, anti-GFAPα and anti-MOG antibodies in the context of a borderline ovarian tumor.

## Case presentation

A 44-year old caucasian woman presented initially with meningism and a history of a flu-like infection including high fever, nausea and vomiting for 4 weeks. On the day of admittance, she experienced temporary confusion, which led her to seek medical attendance. Three weeks prior she had suffered from vaginal herpes infection. No other pre-existing medical conditions were found and the only medication taken were oral contraceptives. There was a negative family history for neurological diseases.

### Clinical findings

The patient developed progressive neurological symptoms over the next 4 days, leading to sopor, psychomotor retardation, paraplegia of the limbs, paresis of the arms (Medical Research Council (MRC) grade 3/5), atactic finger-nose test, gaze-evoked nystagmus and urinary retention. Symptoms progressed to respiratory paralysis necessitating intubation. On the intensive care unit, neurological examination revealed slow pupil response to light, slow corneal reflexes and extinct vestibulo-ocular reflexes, areflexia of the lower limbs and positive palmonental reflexes (see Fig. S1, supplement).

### Diagnostic assessment

**Fig. 1:**
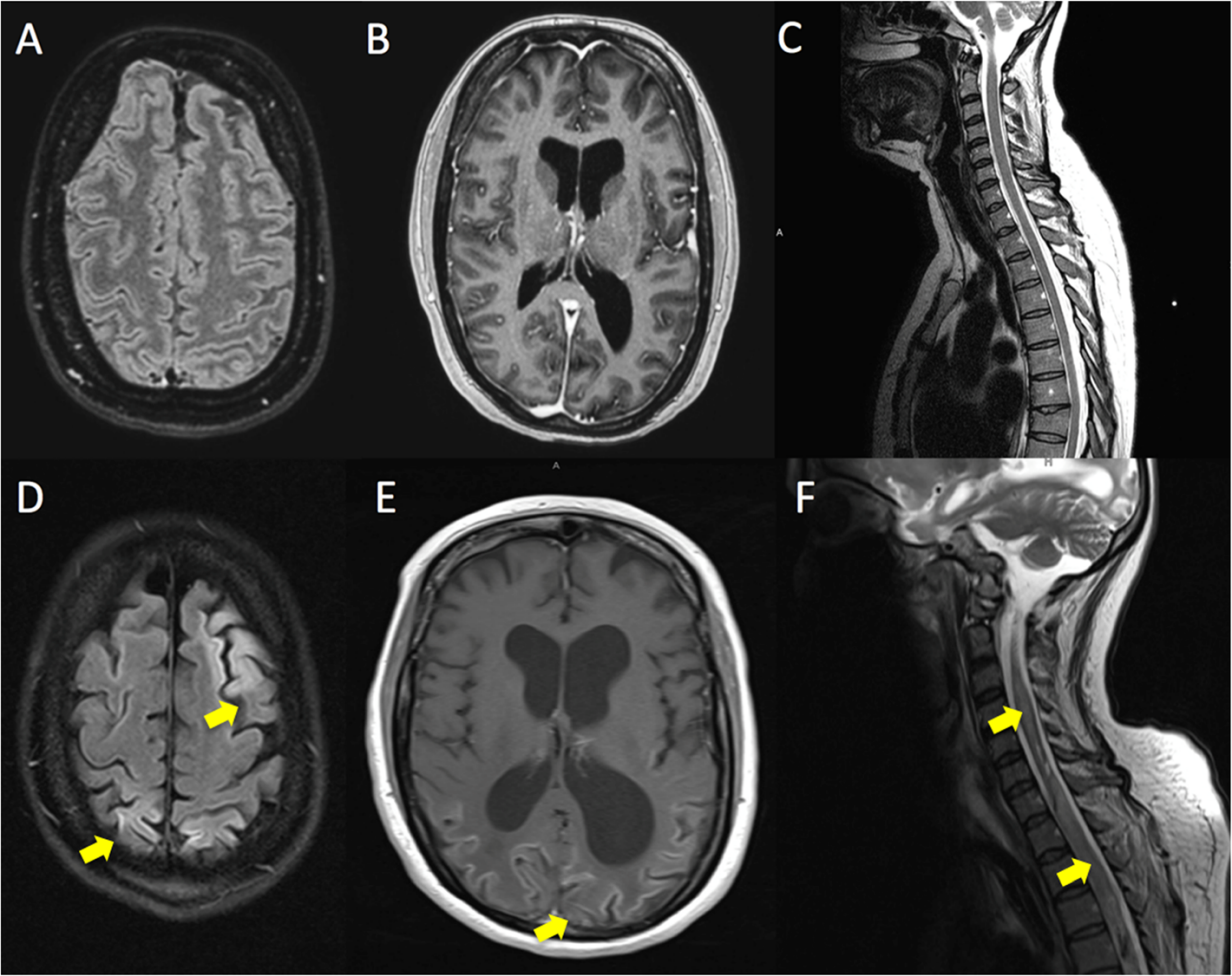
Axial MRI. MRI findings from initial presentation **(A-C, D-F)**. MRI findings of FLAIR **(A, D)**, T1-weighted image with Gd enhancement **(B, E)** and T2w weighted spinal MRI **(C, F)**. Initial MRI of the brain **(A, B)** revealed subtle signs of meningitis, MRI of the spinal cord (C) showed no pathological findings. MRI of the brain after admission to ICU (D, E) revealed cortical and subcortical lesions bihemispherically with parieto-occipital emphasis as well as hyperintense signal alterations of the mesial temporal lobes. MRI of the spinal cord (F) revealed expanded oedema of medulla oblongata. (Marked with yellow arrows)

Initial MRI of the brain revealed signs for meningitis. MRI of the spinal cord showed no pathological findings initially. Later MR imaging of the brain showed diffusion-restricted T2w-hyperintense laminar cortical and subcortical lesions bihemispherically with parieto-occipital emphasis (Fig. S1, supplement), In addition, T2w-hyperintense signal alterations of the mesial temporal lobe and along the tracts up to the upper brainstem and into the medulla oblongata with corresponding DWI restriction were observed. Spinal cord imaging revealed that the edema of the medulla oblongata extended caudally, including almost the entire spinal cord (Fig. 1).

CSF examination demonstrated immune activation and albuminocytologic dissociation (see Table 1) as well as anti-ITPR1, anti-GFAPα and anti-MOG antibodies (Fig. S1, supplement).

**Table 1:** Selected cerebrospinal fluid findings

	Day after presentation at emergency ward			
	1	4	77	104
CSF cell count /μl	58	80	27	32
Total protein mg/l	1336	1815	1538	394
Lactate mmol/l	3.45	4.04	5.87	4.11
Glucose ratio CSF/serum	0.34	0.40	0.14	0.55
Albumin ratio CSF/serum	27.0 × 10^− 3^	49.8	33.2	10.6
L IgG %	0	0	0	0
L IgA %	0	0	0.44	4.56
L IgM %	0	0	0	44.4
Oligoclonal bands	–	Negative	7	5
IgG CSF/serum	–	Negative	Negative	Negative
ITPR1 serum	Negative	Negative	Negative	Negative
ITPR1 CSF	negative	Negative	1:100	Negative
GFAP serum	Negative	Negative	Negative	Negative
GFAP CSF	1:10	1:32	1:10	1:3.2
MOG serum	1:80	1:40	1:40	Negative
MOG CSF	1:2	1:8	Negative	Negative
Microbiological results	IgG Ab index VZV 0.8 (nr: 0.6–1.5), IgG Ab borrelia 0.86 (nr: 0.6–1.5), IgG Ab index HSV 0.7 (nr: 0.6–1.5), Ziehl-neelsen negative, tuberculosis PCR negative, bacterial PCR negative, fungal PCR negative			
Immunological results	NMDA: negative, Aquaporine 4: negative, Amphiphysin Ab negative, CASPR 2 Ab negative, GABA b negative, LGI1 Ab negative, AMPA Ab negative, GAD Ab < 5 IU/ml			

No antibodies were found for: *Mycobacterium tuberculosis*, Treponema pallidum, Borrelia Burgdorferi, Listeria, Leptospires, Tick Borne Encephalitis, HIV, Influenza, Hepatitis B/CCSF-PCR was negative for: eubacterial DNA, panfungal DNA, Herpes simplex virus 1/2*L* Local synthesis of, *Ab* antibody, *nr* normal range

Initially, anti-GFAP and anti-MOG were found positive, during later course anti-ITPR1 was additionally found positive. Anti-ITPR1 and anti-GFAPα were only found in CSF, not in serum, whereas anti-MOG was found both in CSF and serum. Anti-MOG was tested using a life-cell based assay using full length human MOG-EGF tagged as described previously (for details see [5]). Anti-GFAP was tested using human GFAP expressing HEK293T cells and confirmed using primate cerebellum (*Euroimmun*, according to manufacturer’s instructions), as described previously [6, 7]. Anti-ITPR1 was tested using commercially available cell-based assay platforms with fixed cell transfected with human ITPR1 (*Euroimmun*).

Tumor search was performed. CT of thorax and abdomen revealed a tumor in the left ovary. Immediate gynecological presentation followed, and the tumor was removed surgically. Pathological results revealed a borderline tumor (pTis (BT) pR1 V0 L0 Pn0) of micro papillary type. However, the tumor did not express ITPR1, GFAPα or MOG antigen (Fig. S2, supplement). Because of a high likelihood of tumor recurrence, the patient additionally received omentectomy, hysterectomy, contralateral adnexectomy and sample excisions of peritoneum. Pathological results of the second surgery confirmed serous borderline tumor of micropapillary type / non-invasive low-grade serous carcinoma of the right ovary, invasive carcinoma was excluded. Biopsies of peritoneum, ovarian fossa, greater omentum, cervix, portio and endometrium were free of tumor.

Initial neurophysiological analysis showed missing F waves, but normal motor and sensory neurographic findings. Later neurophysiological analyses repeatedly showed missing F waves in all examined nerves, with remaining unremarkable sensory and motor neurographic findings. Somatosensory evoked potentials of median nerve showed non-evoked N13b and N20, P40 of tibial nerve could not be evoked either.

Electroencephalography showed generalized slowing in background rhythm indicative of diffuse cortical dysfunction of initially mild, later moderate degree. Later in the course, left temporo-parietal epileptiform discharges were recorded which disappeared after antiepileptic treatment.

Heart rate variance was pathologically reduced, indicating involvement of the autonomic nervous system.

### Therapeutic intervention

Since the patient presented with a history of flu-like symptoms, fever and meningism and showed a slight pleocytosis in CSF analysis, an infectious meningitis was the initial suspected diagnosis. Therefore, the patient was treated with 3 x 5g ampicillin, 2 g ceftriaxon and 3 x 750mg aciclovir.

After facial myocloni were observed, the patient received additional antiepileptic treatment using levetiracetam 2 x 1g. Since epileptiform discharges were still present in electroencephalogram, antiepileptic treatment was escalated to valproate 3 × 1.5 g and lacosamid 2 x 100mg, until epileptiform discharges and myoclonus disappeared.

Since the patient had progressive neurological symptoms, methylprednisolone 500 mg per day as well as intravenous immunoglobulins 90 g were administered.

No improvements of neurological symptoms were recorded, therefore the patient additionally received rituximab 1000 mg twice within 14 days, as well as 5 sessions of plasma exchange (Fig. S1, supplement).

### Follow-up and outcome

Four months after presentation, the patient was still tetraplegic with areflexia of the lower limbs and positive palmomental reflexes. She still had slow pupil responses to light, with extinct vestibulo-ocular reflexes and slow corneal reflexes. She reacted with grimacing to pain or touch. For aspiration protection, the tracheostoma was left in place, but the patient could breathe without the aid of a ventilation machine. When applying speaking valves, she started to phonate solitary vowels. Due to autonomic involvement, which was proven by pathologic heart rate variance, the patient had several episodes with asystolia, which required cardiac resuscitation at three different time points. In consequence, a cardiac pacemaker was implanted.

### Case and literature review

We report a case of a woman presenting with paraneoplastic encephalomyeloradiculitis associated with a combination of anti-ITPR1, anti-GFAPα and anti-MOG antibodies. The clinical picture suggests phenotypical overlap of all three autoantibodies. Since all autoantibodies were detected in CSF using different and well-established detection techniques, false positive results can be excluded.

While anti-MOG antibodies usually do not occur in association with tumors, both anti-ITPR1-antibodies and anti-GFAPα antibodies have been described as paraneoplastic in around 1/3rd of cases. An expression of antigens corresponding to the found antibodies in the surgically removed tumor cells could not be proven with an additional immunhistological testing. A pure coincidence of antibody (and neurological disease) occurrence and the tumor is therefore possible, but unlikely, considering the existing literature. While the negative finding when testing tumor cells for the relevant antigens at first sight argues against a direct paraneoplastic etiology, it does not exclude an association of the ovarian borderline tumor with the neurological disease. One possible explanation could be that an indirect unspecific activation of the immune system was evoked by the borderline tumor, which indirectly resulted in the production of antibodies. Another explanation is that specific antigen expression was no longer detectable in tumor cells, due to downregulation of anti-tumoral immune response.

To date, no case has been published with paraneoplastic encephalomyeloradiculitis associated with anti-ITPR1, anti-GFAP and anti-MOG. However, one patient [1] has been described positive for three antibodies, including anti-NMDA, anti-ITPR1 and anti-GFAP [1]. This patient initially presented with seizures and encephalopathy, followed 1 week later by opsoclonus myoclonus syndrome. After another 3 weeks, quadriplegia developed due to myelitis. A preceding viral illness was mentioned. Our patient also experienced a preceding vaginal herpes infection. Imaging initially revealed T2 signal changes in medial temporal lobe and right middle cerebellar peduncle, later longitudinal myelitis and lumbar nerve root enhancement was shown (see Table 2). CSF analysis showed pleocytosis (188 leucocytes/μl, protein 127 mg/dl). The patient was treated with steroids, immunoglobulins, rituximab, and plasma exchange. However, the patient remained quadriplegic during the follow-up time of 4 months. Although our case was NMDA-R-IgG negative, the symptoms and outcome of our patient were quite similar.

**Table 2:** Overview of selected clinical and diagnostic findings in the presented case and literature review

	Case	NMDA + ITPR1 + GFAP (i.e. [1])	ITPR1 (i.e. [1])	GFAP (i.e. [6])	MOG (i.e. [8, 9])
**Clinical symptoms**					
Subacute onset	+	+	+	+	+
Prior infection	+ (vaginal herpes)	+		+	+
Meningism	+			+	
Confusion	+	+		+	
Para/Tetraparesis	+	+	+	+	
Sensory loss	+	+	+	+	
Ataxia	+		+	+	
Nystagmus	+		+		
Seizures	+	+	+	+	
Autonomic symptoms	+ (neccessary pace maker)		+	+	
Vision deficits				+	+
Neuropathy			+		
Areflexia	+		+		
**Diagnostic Examinations**					
Signs of meningitis in MRI	+			+	
T2w lesions in cerebral MRI	+ (esp. parieto-occipital)	+ (esp. temporo-cerebellar)		+ (esp. periventricular white matter)	+ (esp. deep grey matter involvement)
Spinal MRI	+ (spinal cord swelling, edema)	+ (long myelitis)	+ (myelitis)	+ (myelitis)	+ (myelitis)
Tumor association	(+) (borderline tumor of left ovary)	-	41% tumor association (no borderline known)	36% tumor association (no borderline known)	Usually no tumor association

To date, 205 cases of anti-GFAPα autoimmunity have been published [6, 7] (see Table S1, supplement). Glial fibrillary acidic protein (GFAPα) is one of the major filament proteins of mature astrocytes. Clinical presentation (see Table 2) is usually subacute, like in our case. Patients typically experience memory loss or confusion, as well as meningeal symptoms (i.e. meningism, headache) and myelopathic symptoms (paresis or hypoesthesia) [7], ataxia and generalized seizures [6]. All listed symptoms were initially present in our case, including temporary confusion, meningism, paresis and hypoesthesia. Typical imaging in patients with anti-GFAP autoimmunity included radial linear periventricular enhancement in cerebral MRI, as well as longitudinal extensive T2 hyperintense lesions in spinal MRI [7]. Tumor association was found in 36% [6, 7], mostly ovarian teratoma. Our case shows a new association with ovarian borderline tumor. Therefore, screening for an underlying tumor should always be performed in GFAP-IgG positive encephalitis cases. Patients with anti-GFAP autoimmunity initially respond well to steroids with a high risk (64%) of relapse during dose tapering [6]. A follow up in 7 cases showed that 6 patients had a bad prognosis, of which 2 died [15].

Myelin oligodendrocyte glycoprotein (MOG) is a membrane protein expressed on the cell surface of oligodendrocytes [12]. Anti-MOG-antibodies are present in atypical demyelinating disorders such as NMOSD. Due to this reason, studies evaluating anti-MOG and NMOSD have been omitted in Table S1 in the supplement, which shows cases similar to our patient, neither suffering from MS, nor NMOSD. Studies evaluating anti-MOG antibodies in children have also been omitted in Table S1 in the supplement. Anti-MOG antibodies have been associated with encephalitis (see Table 2 and Table S1, supplement), similar to our case presentation, leading to potentially life-threatening complications including respiratory impairment [8, 9], while other cases show a less severe course with good treatment response (see Table S1, supplement). Clinical symptoms typically included ataxia and diplopia [2]. The disease was preceded by acute infection or vaccination in 33% [8, 9], similar to our case which was preceded by vaginal herpes infection. Apart from one case with mature teratoma [8, 9], MOG-IgG positivity is rarely found in paraneoplastic contexts. However, many studies do not state that any tumor search was performed (see Table 1).

Inositol 1,4,5-triphosphate receptor type 1 is a protein mediating calcium release from the endoplasmic reticulum [14]. Mutations in the associated gene cause spinocerebellar ataxia type 15 (SCA15) [14]. The most prevalent symptoms for SCA 15 are gait ataxia, dysarthria, nystagmus and limb ataxia [14]. Anti-ITPR1 antibodies were originally described in patients with autoimmune cerebellar ataxia [10, 11]. A more recent publication suggested a broader disease spectrum (see Table 2), including motor, sensory and autonomic symptoms [8, 9]. In fact, peripheral neuropathy appears to be just as common as cerebellar ataxia. Peripheral neuropathy was also found to be most commonly associated with malignancy [8, 9]. These symptoms may be explained, since anti-ITPR1 is not only expressed in the brain, but also in the peripheral nervous system [4]. It is also expressed in the autonomic nervous system, especially in the sympathetic ganglia [4], possibly explaining pathological heart rate variety of our patient. Reduced pathological heart frequency variability and later repeated stroke episodes attributed to intermittent atrial fibrillation was also detected in one other case [8, 9]. Interestingly, ITPR receptors have recently been shown to be present in murine cardiac myocytes, modulating intracellular calcium pathways [13], which could explain arrhythmia. Twenty-five other cases of anti-ITPR1 autoimmunity have been published to date [1, 3, 8, 9, 11] (see Table S1, supplement). Tumor association was present in 41% (10 out of 23, in 3 cases no tumor search was performed), including our case associated with ovarian borderline tumor, as well as four cases linked to breast cancer [1, 3] (one of them also had endometrial carcinoma [1]), one case with cervical dysplasia [1], one case with non-Hodgkin lymphoma [8, 9], two cases associated with lung cancer [1, 8, 9] and another case that was linked to renal cell carcinoma metastatic to bones and liver at time of neurologic presentation [1]. Due to this association with tumors, we strongly recommend a thorough search for neoplasia, whenever anti-ITPR1 antibodies are detected. In one case, breast cancer diagnosis was made 11 years after onset of cerebellar syndrome [3], therefore even when no neoplasia can be found, follow-up examinations are recommended. Both treatment type (e.g., intravenous steroids and immunoglobulins, plasma exchange and rituximab) and treatment outcome varied widely over the known cases. It is therefore impossible to predict outcome to date.

## Conclusions

In conclusion, we present a review of the literature as well as a novel case of paraneoplastic encephalomyeloradiculitis associated with a combination of anti-ITPR1, anti-GFAP and anti-MOG antibodies. It is tempting to speculate that immune responses might have been triggered by a borderline ovarian tumor, which has not been described in association with either of these antibodies so far. However, these antibodies may also have occurred as a consequence of tissue destruction.

## Supplementary Information

**Additional file 1.**

## Data Availability

Not applicable.

## Abbreviations

ADEM: Acute disseminated encephalomyelitis
AMPA: Alpha-amino-3-hydroxy-5-methyl-4-isoxazolepropionic acid
AQP4: Aquaporine 4
CSF: Cerebrospinal fluid
CT: Computer tomography
CASPR 2: Contactin-associated protein 2
DWI: Diffusion weighted Imaging
EGF: Epidermal growth factor
EAE: experimental autoimmune encephalomyelitis
GABA: Gamma-aminobutyric acid
GFAP: Glial-fibrillary-acidic-protein
GAD: Glutamic acid decarboxylase
Ig: Immunglobulin
ITPR1: Inositol 1,4,5-triphosphate receptor type 1
IFN: interferon
LGI1: Leucine-rich, glioma Inactivated 1
MRI: Magnetic Resonance Imaging
MRC: Medical Research Council
MS: Multiple sclerosis
MOG: Myelin oligodendrocyte glycoprotein
NMDA: N-Methyl-D-Aspartat
NME: Necrotizing meningoencephalitis
NMOSD: Neuromyelitis optica spectrum disorder
SCA: spinocerebella ataxia
