# Supplementary material for: Paraneoplastic encephalomyeloradiculits with multiple autoantibodies against ITPR-1, GFAP and MOG: case report and literature review
Source: Neurol Res Pract. 2021 Oct 11;3:48. doi: 10.1186/s42466-021-00145-w (PMC8504129; doi:10.1186/s42466-021-00145-w)
Supplement: Supplementary file 1 — Additional file 1. [file 42466_2021_145_MOESM1_ESM.docx]

**Supplementary material**

Figure S1: Timeline of initial presentation, clinical symptoms, treatment, imaging and surgery


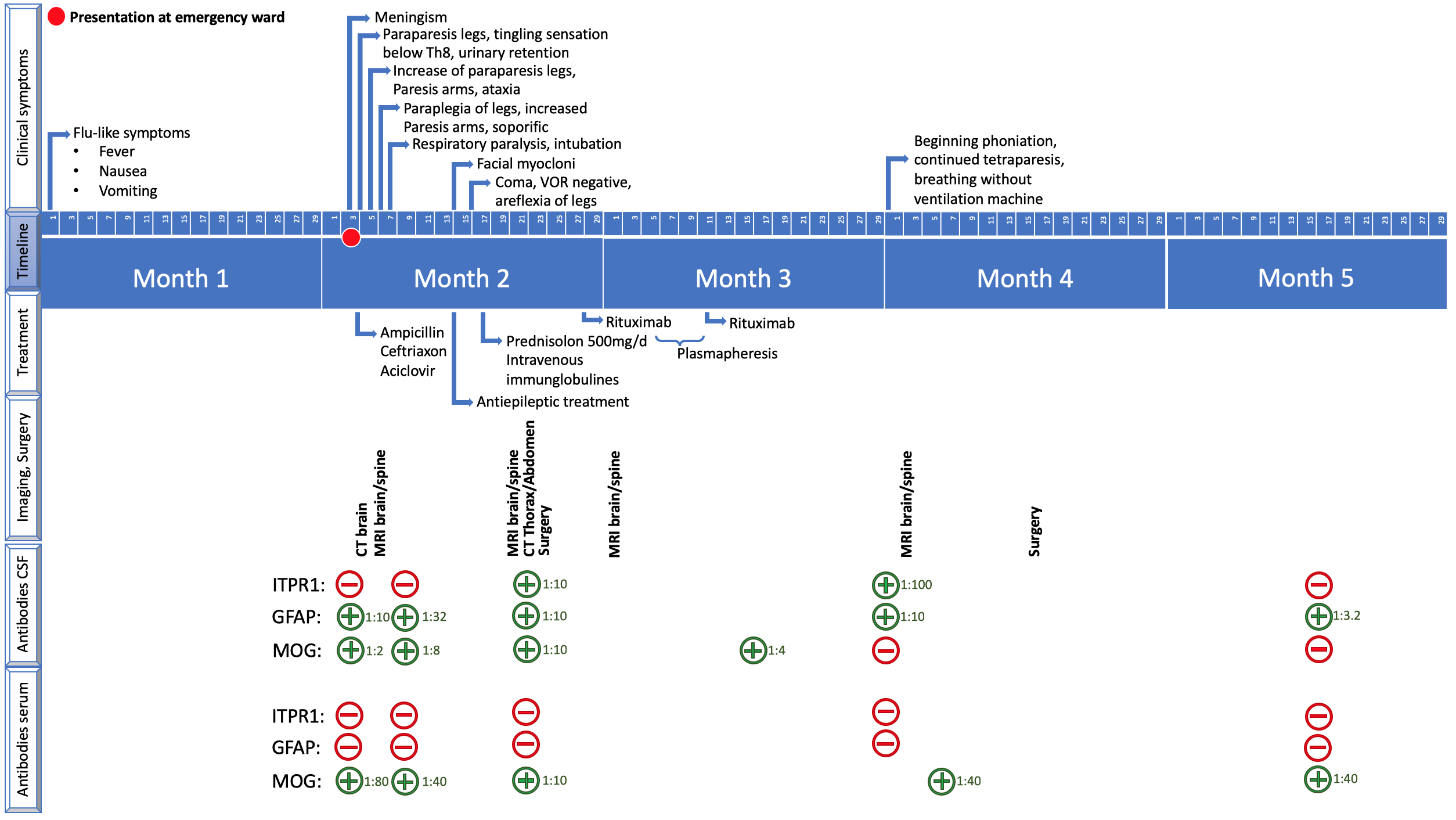


Figure S2: Immunohistochemistry on formalin-fixed and paraffin embedded tumor tissue of the patient is negative for anti-ITPR1 (A; p.c. rabbit antibody; Dianova), anti-GFAP (B; p.c. rabbit antibody; DAKO), and anti-MOG (C; m.c. mouse antibody; Sigma Aldrich). Scale bars: 30 μ.


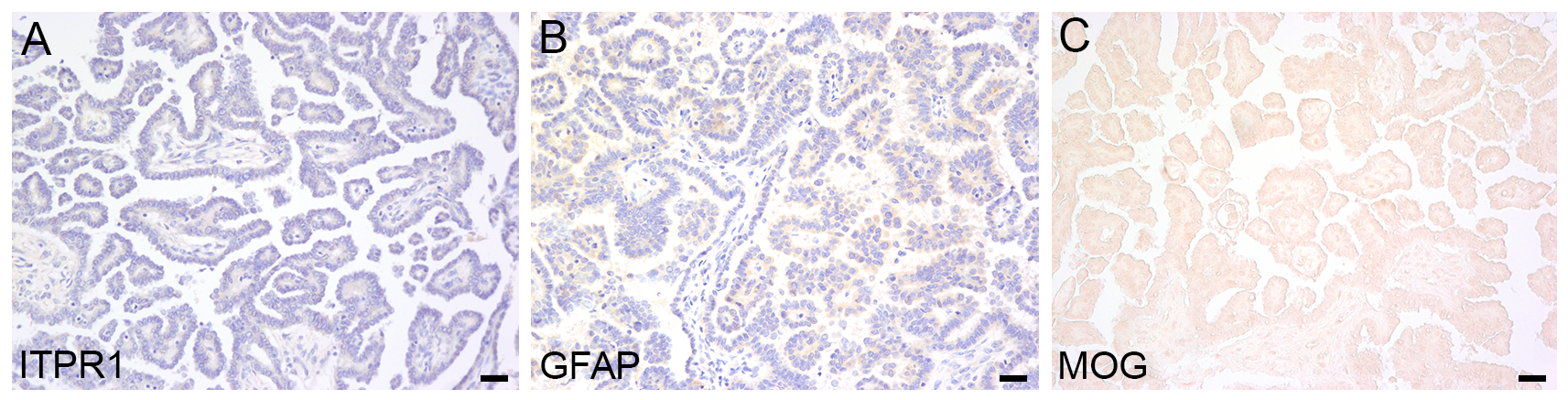


Table S1: Studies concerning cases with GFAP and ITRP1 antibody positive encephalitis

| **Author** | **n** | **Age at onset** | **Antibody** | **Clinical symptoms** | **Radiology** | **CSF** | **Treatment** | **Symptom improvement** | **Associated neoplasia** | **Neurophysiology** |
| --- | --- | --- | --- | --- | --- | --- | --- | --- | --- | --- |
| Flanagan, 2017 | 102 | 44 | GFAP | Meningism, encephalitis, myelitis, neuropathy, ataxia, epilepsy, dysautonomia | Perivascular enhancement. Spinal: longitudinally extensive myelitic lesions | 94% inflammatory | High dose corticosteroids, immuno-suppression | 6% prolonged corticosteroid response. Requirement for immuno-suppression | 35/102 patients (34%) | - |
| Fang, 2016 | 103 | 42 | GFAP | Headache, subacute encephalopathy, optic papillitis, inflammatory myelitis, postural tremor, cerebellar ataxia | Linear perivascular enhancement | 93% inflammatory | High dose corticosteroids, immuno-suppression | Tendency towards relapse without long-term immuno-suppression | 36/103 patients (38%) | - - |
| Alfugham, 2018 | 14 | 64 | ITPR1 | Peripheral neuropathy, cerebellar ataxia, sensory ataxia, encephalitis with seizures, subacute progressive axonal sensory and later motor polyneuropathy, myelopathy with proprioceptive loss. Quadriplegia. Myoclonus, even Opsoclonus myoclonus syndrome, pandysautonomia, cognitive decline, vertigo, dysphasia, agraphia, nystagmus, nausea, double vision, hearing loss, visual blurring | Spinal: diffuse enhancement of cervical or lumbar nerve roots, longitudinally extensive increased T2 signal, long myelitis  Brain: generalized brain atrophy, cerebellar atrophy, subcortical right occipito-parietal high T2 signal with contrast enhancement  PET: increased uptake in right cerebellum | Elevated protein levels in 5 patients, pleocytosis in 4 patients. In one patient additionally positive for NMDA-R-IgG and GFAP-IgG (this patient had preceding viral illness) | High dosage of steroids, plasma exchange, IVIG, Rituximab | 1 patient had Initial good improvement, but later progressed, 2 patients did not benefit, 4 died, 3 mild benefit, 2 no benefit | 5/14 patients (36%) | EEG showed unilateral periodic discharges. EMG showed demyelinating peripheral neuropathy, diffuse axonal neuropathy |
| Berzero, 2017 | 1 | 31 | ITPR1 | Progressive ataxia, mild dysarthria. Over the next 2 years unable to walk unassisted | Ponto-cerebellar atrophy, hot cross bun sign | Acellular, normal glucose and protein levels, no oligoclonal bands | 6 cycles high dose methyl-prednisolone, 10 sessions plasma exchange. Later again 3 cycles of immunoglobulin 2g/kg | No clinical improvement | 1/1 patients (100%) | Neurophysiology: No peripheral neuropathy |
| Jarius, 2016 | 3 | 60 | ITPR1 | Radiculo-polyneuropathy, motor, sensory and autonomic symptoms. | Unremarkable of brain and spine | Albumin-cytologic dissociation, systemic immune activation | Plasma exchange (7x), intravenous immunoglobulines (5x25g) | Initially no significant clinical improvement, after 4 months’ improvement of paresis | 2/3 patients (67%) | Neurophysiology: Missing f waves, a waves, delayed motor nerve conduction velocities, decreased sensory nerve action potentials. Reduced heart rate variability |
| Fouka, 2016 | 3 | 54 | ITPR1^1^ | Gait imbalance, subacute walking impairment, horizontal nystagmus, bilateral intention tremor, dysarthria, dysmetria, dysdiadochokinesis, dysautonomia (orthostatic hypotension), REM sleep disturbances | Diffuse non-enhancing demyelinating lesions, cerebella atrophy, multiple lesions of ischemic origin | Elevated IgG index, no signs of inflammation | steroids | Symptoms progressed, no treatment benefit | - ^2^ |  |
| Jarius, 2014 (8), 2015 | 4 | - | ITPR1 | Cerebellar ataxia, chronic cerebellar syndrome, dysarthria, gaze disturbance | Moderate cerebellar atrophy | - | Responsive to immunotherapy. One patient received 10 cycles of plasma exchange | One patient was responsive to immunotherapy. One patient had extensive disease progression. Other patients not stated. | 0/4 patients (0%) | - |
| Berzero, 2018 | 1 | 43 | MOG | Gait ataxia, mild dysarthria, nystagmus, right hypoesthesia. Gradually deteriorated to dysphagia, diplopia, unable to walk unassisted | Large confluent ponto-mesencephalic fluid-attenuated inversion recovery (FLAIR) hyperintensities surrounding fourth ventricle and cerebral aqueduct, with punctuate and linear contrast enhancement after gadolinium injection. Spinal MRI normal. PET normal. McDonald criteria for MS not met. | Normal protein levels and cell count, presence of CSF restricted oligoclonal bands | High dose steroids. After relapse azathioprine | Dramatic clinical and radiological response. Complete recovery except residual mild ataxia. However, 4 months after steroid discontinuation clinical relapse. After azathioprine, favorable clinical response. | - ^2^ |  |
| Mariotto, 2017 | 1 | 31 | MOG | Altered mental status, fever, ataxia, unsteady gate, reduced level of consciousness | Lesions found subcortical, periventricular, cortical, white matter, thalamus, basal ganglia, internal capsule, pons | Pleocytosis, increased protein | Intravenous immunoglobulins, aciclovir, antibiotics | Clinical and radiological improvement leading to almost full recovery. No relapse. | - ^2^ |  |
| Fujimori, 2017 | 1 | 46 | MOG | Dizziness, seizures, optic neuritis | Lesions found in frontal cortex, corpus callosum, cingulate gyri, frontal lobes, aqueduct, thalamus | pleocytosis | Methylprednisolone, acyclovir, oral prednisolone | Initial full recovery, however, with relapses | - ^2^ |  |
| Ogawa, 2017 | 4 | 37 | MOG | Headache, seizures, visual loss, dysuria, disorientation, agitation, emotional incontinence, aphasia, hemiparesis | Lesions in optic nerve, cortex, in 2 patients unremarkable. Hyperperfusion on SPECT. | Pleocytosis | Methylprednisolone, anti-epileptics, acyclovir, antibiotics, antimycotics, dexamethasone | Full recovery | - ^2^ |  |
| Jarius, 2016 | 50 | 31 | MOG | Optic neuritis and/or myelitis, 30% brainstem encephalitis with: respiratory insufficiency, nausea, vomiting, dysarthria, dysphagia, impaired cough reflex, oculomotor nerve palsy, diplopia, nystagmus, internuclear ophtalmoplegia, facial nerve paresis, trigeminal hypesthesia/dysesthesia, vertigo, hearing loss, balance difficulties, gait and limb ataxia. | Lesions in pons, medulla oblongata, mesencephalon, cerebellar peduncles, adjacent to fourth ventricle, periaqueductal grey, diencephalic or cerebellar lesions. | Pleocytosis in 79%. oligoclonal bands in 29%. All negative for AQP4-IgG. | Steroids. Plasma exchange. mitoxantrone, azathioprine, rituximab, natalizumab, IFN-beta and IVIG | Relapsing course in 87%. Brainstem involvement associated with more aggressive general disease course (higher relapse rate, more myelitis attacks, more supratentorial brain lesions, worse EDSS) | 1 teratoma, 1 ganglioneuroma (2/50, 4%) | Disease preceded by acute infection or vaccination in 33% |
| Dalmau, 2017 | 3 | 23-39 | MOG | epilepsy | Unilateral cortical lesions | Pleocytosis. NMDA negative, AQP4 negative | Steroids | recovery | - ^2^ |  |
| Yamamoto, 2017 | 1 | 29 | MOG | Headache, fever. Later right arm tremor. | Increased signals in left cerebral cortex and DWI | Pleocytosis, elevated protein | Methylprednisolone | Rapid recovery | - ^2^ |  |
| Fujimori, 2017 | 1 | 46 | MOG | Dizziness initially, later focal seizure in right leg which later generalized. Over the next week headache, paraparesis. Progression to paraplegic with spasticity, fever, memory decline, lethargy | Lesion in left frontal cortex, later also of corpus callosum, bilateral cingulate gyri, bilateral frontal lobes. Spinal MRI normal | Pleocytosis, normal protein and glucose. NMDA negative. | High dose intravenous methylprednisolone, aciclovir | Initial symptom improvement leading to walking without assistance. Later relapse with right optic neuritis. | - ^2^ | No epileptic discharges in EEG |
| Spadaro, 2014 | 1 | 66 | MOG | Leg weakness and hypoesthesia below Th7. Nausea, dysphagia, double vision, dysarthria, left sided facial paresis | T2 hyperintense lesion Th8/9. Scattered non-specific white matter lesions in cerebral MRI | Normal. NMDA negative, AQP4 negative | Immunoadsorption, immunoglobulins, rituximab | Initial marked clinical improvement, however, relapses occurred | - ^2^ | Visual evoked potentials normal |
| Banks, 2020 | 185 | 24 | MOG | 63% were symptomatic, typical symptoms included ataxia and diplopia, nausea, dysarthria, vertigo | Diffuse middle cerebellar peduncle MRI lesions, diffuse medulla, pons or midbrain MRI lesions | Oligoclonal bands were rare | Intravenous steroids, plasma exchange, intravenous IG, rituximab, azathioprine, mycophenolate | Majority responded well to immunotherapy | - ^2^ |  |

NA=not applicable, ITPR1=inositol 1,4,5-triphosphate receptor type 1, GFAP=glial fibrillary acidic protein, ADEM=acute disseminated encephalomyelitis, CSF=cerebrospinal fluid, NP=neurophysiology, *not applicable, since study is a review. ^1^in the article referred to as IP3R1, also known as ITPR1. ^2^no tumor search performed/mentioned
